# Lung type 3 innate lymphoid cells respond early following *Mycobacterium tuberculosis* infection

**DOI:** 10.1128/mbio.03299-23

**Published:** 2024-02-26

**Authors:** Shibali Das, Kuldeep Singh Chauhan, Mushtaq Ahmed, Sadia Akter, Lan Lu, Marco Colonna, Shabaana A. Khader

**Affiliations:** 1 Department of Molecular Microbiology, Washington University in St. Louis, St. Louis, Missouri, USA; 2 Department of Microbiology, University of Chicago, Chicago, Illinois, USA; 3 Department of Pathology and Immunology, Division of Immunobiology, Washington University School of Medicine, St. Louis, Missouri, USA; National Institute of Allergy and Infectious Diseases, Bethesda, Maryland, USA

**Keywords:** *Mycobacterium tuberculosis*, innate lymphoid cells, innate immunity, epithelial cells, tuberculosis vaccines

## Abstract

Tuberculosis is the leading cause of death due to an infectious disease worldwide. Innate lymphoid type 3 cells (ILC3s) mediate early protection during *Mycobacterium tuberculosis* (*Mtb*) infection. However, the early signaling mechanisms that govern ILC3 activation or recruitment within the lung during *Mtb* infection are unclear. scRNA-seq analysis of *Mtb*-infected mouse lung innate lymphoid cells (ILCs) has revealed the presence of different clusters of ILC populations, suggesting heterogeneity. Using mouse models, we show that during *Mtb* infection, interleukin-1 receptor (IL-1R) signaling on epithelial cells drives ILC3 expansion and regulates ILC3 accumulation in the lung. Furthermore, our data revealed that C-X-C motif chemokine receptor 5 (CXCR5) signaling plays a crucial role in ILC3 recruitment from periphery during *Mtb* infection. Our study thus establishes the early responses that drive ILC3 accumulation during *Mtb* infection and points to ILC3s as a potential vaccine target.

**IMPORTANCE:**

Tuberculosis is a leading cause of death due to a single infectious agent accounting for 1.6 million deaths each year. In our study, we determined the role of type 3 innate lymphoid cells in early immune events necessary for achieving protection during *Mtb* infection. Our study reveals distinct clusters of ILC2, ILC3, and ILC3/ILC1-like cells in *Mtb* infection. Moreover, our study reveal that IL-1R signaling on lung type 2 epithelial cells plays a key role in lung ILC3 accumulation during *Mtb* infection. CXCR5 on ILC3s is involved in ILC3 homing from periphery during *Mtb* infection. Thus, our study provides novel insights into the early immune mechanisms governed by innate lymphoid cells that can be targeted for potential vaccine-induced protection.

## INTRODUCTION

Tuberculosis (TB), caused by the bacterium *Mycobacterium tuberculosis* (*Mtb*), infects approximately one-fourth of the world’s population, resulting in 1.6 million deaths in 2021 globally (1). In 2021 alone, an estimated 10.6 million people fell ill with TB. A major hurdle to eradicate the disease is our poor understanding of the early immune mechanisms mediating protective immunity against *Mtb* infection.

In recent studies, innate immunity has gained importance in controlling *Mtb* infection prior to or despite the engagement of adaptive immune responses (2–5). Our own recent study has established a protective role for innate lymphoid cells (ILCs) in the lungs of mice challenged with *Mtb* (2). As another important arm of innate immunity, ILCs share features with both adaptive and innate immune cells and comprise three main subsets. Group 1 ILCs produce interferon γ (IFN-γ) and include natural killer (NK) cells and non-cytotoxic, non-NK type 1 ILCs, which are related to type 1 immune response (Th1) (6). Group 2 ILCs, which produce interleukin-4 (IL-4), IL-5, and IL-13, are involved in inflammatory-linked airway hyperactivity, tissue repair (7), and helminth clearance by promoting type 2 immune responses (8). Innate lymphoid type 3 cells (ILC3s) produce IL-17 and/or IL-22 (related to Th17 immune response) (9) and can participate in the strategic positioning of immune cells in ectopic lymphoid structures (10). ILCs are also critical for tissue repair of the lungs following infection-induced lung damage (11) and for generating hepatic granulomas (12). Indeed, our recent study reported that ILC3 mediated early protection against *Mtb* through enhanced secretion of IL-17 and IL-22 (2). However, not much is known about the early signaling mechanisms that govern ILC3 accumulation and control of *Mtb* infection.

In the current study, we show that ILC3s are early responders upon exposure to *Mtb*. During early infection, we demonstrate that interleukin-1 receptor (IL-1R) signaling plays a crucial role in regulating lung ILC3 accumulation, formation of granulomas, and *Mtb* control. Depletion of IL-23, together with IL-1R signaling, results in increased susceptibility to *Mtb* infection, with reduced accumulation of lung ILC3 subsets and myeloid cells, and loss of *Mtb* control. Importantly, following *Mtb* infection, IL-1R signaling within the epithelial cells provides critical signals for CXCR5^+^ ILC3 accumulation, myeloid cell accumulation, and early control of *Mtb* replication. Taken together, our data provide novel evidence for an early signaling mechanism in mediating ILC3 localization and immunity against emerging *Mtb* infections.

## RESULTS

### *Mtb* infection induced early accumulation of ILC subsets

**Fig 1:**
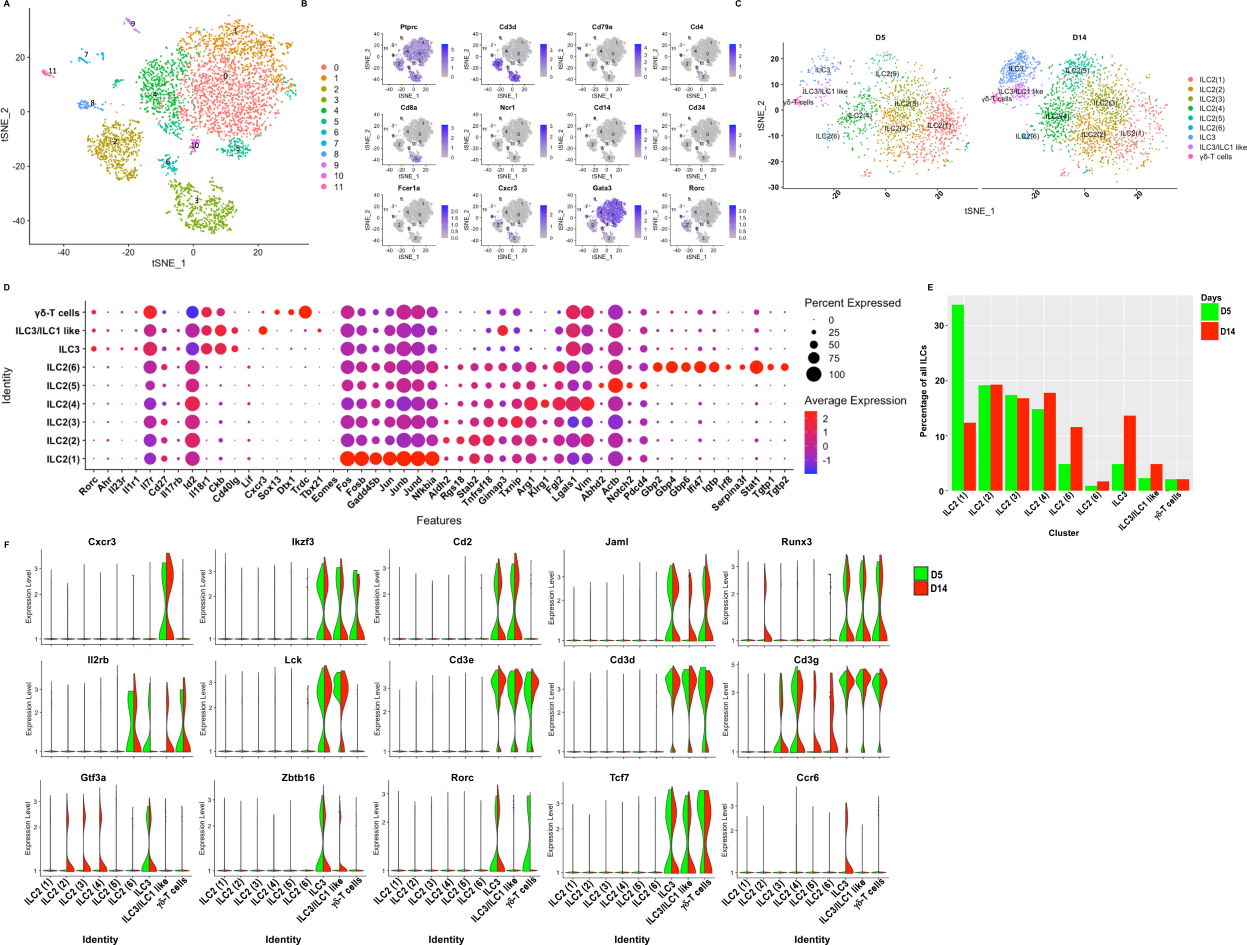
scRNA-seq transcriptional profiling of lung ILCs isolated following *Mtb* infection. B6 mice were aerosol infected with *Mtb* HN878, and lungs were harvested at 5 and 14 dpi. (**A**) t-Distributed stochastic neighbor embedding (tSNE) visualization of the different clusters (all samples together). (**B**) tSNE plot with the expression of known cell markers. The expression of marker genes was used to characterize distinct clusters according to cell identity. (**C**) tSNE plot of ILC subtypes after re-clustering the ILCs only, colored according to cellular identity, split by days. (**D**) Dot plot indicating expression of key genes detected for each cell subtype. The dot color represents the expression level, and the dot size represents the percentage of cells in each cluster expressing a particular gene. (**E**) Distribution of ILC subsets by cluster per days (infected at 5 dpi, pooled from 10 mice; infected at 14 dpi, pooled from 10 mice). (**F**) Violin plot of genes in ILC3, ILC3/ILC1-like and γδ T-cell subsets by cluster per days (infected at 5 dpi, pooled from 10 mice; infected at 14 dpi, pooled from 10 mice).

During *Mtb* infection, lung ILC populations, specifically ILC3s, increased early following *Mtb* infection in the murine lung (2). Therefore, to gain better insight about the functional heterogeneity of lung ILCs, we characterized them at the single-cell level following *Mtb* infection. We sorted for highly pure innate lymphoid cells (CD45^+^CD127^+^Lin^−^NK1.1^−^) from *Mtb*-infected lung at 5 and 14 days post-infection (dpi) and performed scRNA-seq. Following single-cell alignment and quality control process, we found a total of 5,084 high-quality cells (1,886 cells in 5 dpi and 3,198 cells in 14 dpi). Cluster analysis found 12 distinct clusters (Fig. 1A) consisting of 78.9% of total cells, with six clusters of ILC population in addition to other types of cells. The identification of the cell types is based on the expression of known markers (Fig. 1B). Clusters 0, 1, 4, 5, and 10 are ILC2 (*Gata*^+^); cluster 2 is mix of ILC1 (*Cxcr3^+^*), ILC2 (*Gata*^+^), and ILC3 (*Rorc^+^*); cluster 3 is T cells (*Cd3d*^+^, *Cd4*^*+*^, and *Cd8a*^+^); cluster 6 is dendritic cells; cluster 7 is an unknown type of cells; cluster 8 is NK (*Ncr1*^+^) cells; cluster 9 is dendritic macrophage (*Cd14*^+^); and cluster 11 is B (*Cd79a*^+^) cells (Fig. 1B).

To better understand the ILC population, we next performed re-clustering analysis of the ILCs (clusters 0, 1, 2, 4, 5, and 10) with the highest possible resolution (resolution = 1.0) using Seurat and revealed a total of nine clusters: six ILC2 clusters (*Gata^+^*), one ILC3 cluster (*Rorc*^+^), one ILC3/ILC1-like cluster (*Rorc*^+^ and *Cxcr3*^+^), and one γδ T (*Trdc*^+^ and *Sox13*^*+*^) cell cluster (Fig. 1C; Fig. S2A).

As expected, ILC2 cells were well represented (11) in the lung and expressed high level of inhibitor of DNA binding 2 (*Id2*) and low level of *Il7r* (Fig. 1E). Most of the ILC2 clusters [ILC2 (1), ILC2 (2), ILC2 (3), and ILC2 (6)] expressed *Cd27*. In one of the ILC2 clusters, ILC2 (1), there was drastically reduced frequency of cells in the infected lung at 14 dpi (12%) compared to 5 dpi (34%) (Fig. 1C and E). ILC2 (1) expresses Fos gene family: FBJ osteosarcoma oncogene (*Fos* and *Fosb*), Jun proto-oncogenes (*Jun*, *Junb*, and *Jund*), and nuclear factor of kappa light polypeptide gene enhancer in B-cell inhibitor, alpha (*Nfkbia*) (Fig. 1D). However, cluster ILC2 (2) expresses aldehyde dehydrogenase 2 (*Aldh2*), regulator of G-protein signaling 18 (*Rgs18*), stabilin 2 (*Stab2*), and tumor necrosis factor receptor superfamily, member 18 (*Tnfrsf18*). On the other hand, cluster ILC2 (3) had higher expression of GTPase, IMAP family member 3 (*Gimap3*), and thioredoxin interacting protein (*Txnip*); however, cluster ILC2 (4) expressed arginase (*Arg1*), fibrinogen-like protein 2 (*Fgl2*), killer cell lectin-like receptor subfamily G, member 1 (*Klrg1*), and vimentin (*Vim*). Cluster ILC2 (5) highly expressed actin-beta (*Actb*), in addition to *Notch2* and programmed cell death 4 (*Pdcd4*). The last ILC2 cluster, ILC2 (6), highly expressed many IFN genes, guanylate binding proteins 2, 4, and 6 (*Gbp2*, *Gbp4*, and *Gbp6*), interferon gamma inducible protein 47 (*Ifi47*), interferon gamma induced GTPase (*Igtp*), interferon regulatory factor 8 (*Irf8*), signal transducer and activator of transcription 1 (*Stat1*), T cell-specific GTPase 1 and 2 (*Tgtp1* and *Tgtp2*), and serine (or cysteine) peptidase inhibitor, clade A, member 3F (*Serpina3f*) (Fig. 1D).

We observed accumulation of ILC3 and ILC3/ILC1-like clusters in the infected lung at 14 dpi compared to 5 dpi (Fig. 1C). Both the ILC3 cluster and the ILC3/ILC1-like cluster expressed *Il7r*, *Il18r1*, creatine kinase B (*Ckb*), and Cd40 ligand (*Cd40lg*), in addition to *Rorc*, *Ahr*, *Il23r*, *Il1r*, and *Il17rb*. However, the ILC3/ILC1-like cluster also expressed *Cxcr3* and *Tbx21* (Fig. 1D). On the other hand, the γδ T-cell cluster, which co-purified with the ILC sort, expressed higher levels of *Il7r* and *Trdc*, and moderate level of *Il18r*, while about 25% of the cells expressed a high level of *Sox13* and deltex E3 ubiquitin ligase 1. Indeed, we observed an accumulation of ILC3 cells, from 5% of the total ILC cells in the infected lungs at 5 dpi to 14% in the infected lung at 14 dpi (Fig. 1E). Moreover, ILC3/ILC1-like cluster also increased from 2% at 5 dpi to 5% at 14 dpi (Fig. 1E).

Based on the gene expression profile, we observed increased and consistent expression of IKAROS family zinc finger 1 (*Ikzf3*), CD2, junction adhesion molecule like (*Jaml*), RUNX family transcription factor 3 (*Runx3*), lymphocyte-specific protein tyrosine kinase (*Lck*), *Cd3e*, *d,* and *g*, zinc finger and BTB domain containing 16 (*Zbtb16*), *Rorc*, transcription factor 7 (*Tcf7*) within ILC3, ILC3/ILC1-like cells, and γδT cell clusters (Fig. 1F) over the time course we measured. Additionally, we observed either increased or consistent expression of interleukin-1 receptor-like 1 (*Il1rl1*), CC motif chemokine receptor 8 (*Ccr8*), *Klrg1*, *annexin A1* (*Anxa1*), *Tnfrsf18*, *CD81*, and *arginase 1* (*Arg1*) within the ILC2 clusters. Moreover, we observed consistent expression of *Il5* only in ILC2 (4) subsets, peroxisome proliferator-activated receptor gamma (*Pparg*) in ILC2 (2) at 5 dpi, AT-rich interaction domain 5B (*Arid5b*) and amphiregulin (*Areg*) in ILC2 (1) and ILC2 (6), early growth response 1 (*Egr1*) in ILC2 (1), C-C motif chemokine ligand 5 (*Ccl5*) in ILC2 (4) and ILC2 (6), *Kit* in ILC2 (1), ILC2 (2), and ILC2 (4), and finally TNF superfamily member 11 (*Tnfsf11*) in ILC2 (2) at both time points and ILC2 (6) at 14 dpi (Fig. S2B).

Additionally, we observed consistent gene expression in most of the ILC subsets over the two time points tested. The gene list included Kruppel-like factor 6 (*Klf6)*, lectin, galactose binding, soluble 1 (*Lgals1*), *lamin A/C (Lmna)*, *DEAD-box helicase 5*, *Cd69*, *Vim*, *CXC motif chemokine receptor 6 (Cxcr6)*, and *lymphotoxin beta* (*Ltb*) (Fig. S2C). However, we could also detect constantly high expression of B-cell lymphoma/leukemia 11B (*Bcl11b*), musculoaponeurotic fibrosarcoma (*Maf*), and inducible T-cell co-stimulator (*Icos*) within the ILC3, ILC3/ILC1-like cells, and γδT cell clusters. On the other hand, we could detect higher expression of Kruppel-like factor 10(*Klf10)* and GATA binding protein 3 *(Gata3*) within the ILC2 clusters.

Therefore, our single-cell transcriptomic analysis not only confirmed the early and rapid accumulation of ILC3 and ILC3/ILC1-like clusters in the *Mtb*-infected lungs but also provided important information about the gene expression profiles within the clusters that changed over the course of infection.

### IL-1/IL-23 cytokine axis regulates the accumulation of ILC3s during *Mtb* infection

**Fig 2:**
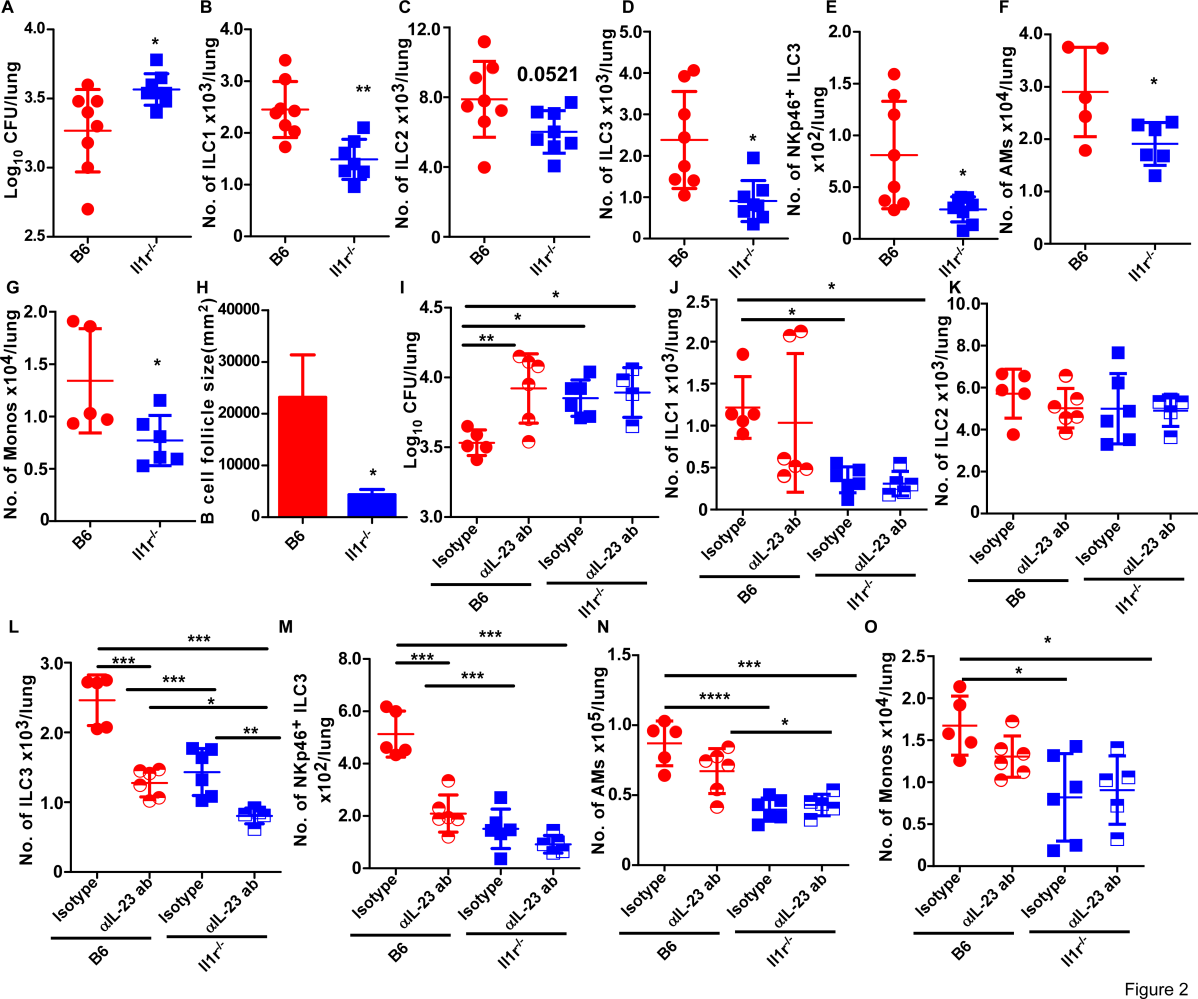
IL-1β/IL-23 cytokine axis regulates the early accumulation of ILC3s during *Mtb* infection. *Il1r*^−/−^ and B6 mice were aerosol-infected with 100 CFU *Mtb* HN878. (**A**) Lung bacterial burden at 14 dpi was determined by plating (*n* = 5–8 per group). (**B–E**) Numbers of ILC1, ILC2, ILC3s, and NKp46^+^ ILC3s were determined by flow cytometry; (**F**) alveolar macrophages (AMs) and (**G**) monocytes were measured by flow cytometry on total lung single-cell suspensions at 14 dpi. (**H**) Formalin-fixed paraffin-embedded lung sections from *Mtb*-infected mice were stained with antibodies against B220, and the average sizes of B-cell follicles were quantified. In a separate experiment, C57BL/6 and *Il1r*^−/−^ mice received IL-23 neutralizing antibody or isotype control antibody (by intraperitoneal injection) 1 day before infection with around 100 CFU *Mtb*, and the lung bacterial burden was determined by plating (**I**), and numbers of ILC1s, ILC2s and ILC3s, NKp46^+^ ILC3s, AMs, and monocytes (**J–O**) were quantified at 14 dpi using flow cytometry (*n* = 5–6 mice for isotype, *n* = 5–6 mice for anti-IL23 antibody). All data are mean ± SD. *P ≤ 0.05, **P ≤ 0.01, ***P ≤ 0.001, ****P ≤ 0.0001 were determined by either Student’s *t*-test (A–H) or one-way analysis of variance (I–O).

Lung scRNA-seq data revealed expression of *Il1r* and *Il23r* in the *Mtb*-infected lung ILC3 cluster. Therefore, we next determined the importance of IL-1R and IL-23R signaling in the accumulation of ILC populations following *Mtb* infection. As known, the absence of IL-1R signaling (*Il1r^−^*^/^*^−^*) in mice resulted in increased susceptibility due to *Mtb* infection (13) (Fig. 2A). Moreover, this increased *Mtb* susceptibility was also associated with reduced accumulation of lung ILC1s, ILC3s, and NKp46-expressing ILC3s (NKP-ILC3s) at 14 dpi (Fig. 2B, D and E; Fig. S1C and D). However, the accumulation of ILC2s was not impacted in the *Mtb*-infected *Il1r^−/−^* mice (Fig. 2C). Importantly, the reduced ILC3 accumulation coincided with reduced accumulation of alveolar macrophages (AMs) and monocytes in the *Mtb*-infected *Il1r^−/−^* mice lung at 14 dpi (Fig. 2F and G; Fig. S1B). These changes in myeloid cell accumulation were also reflected in poorly formed granuloma-associated lymphoid tissue (GrALT) (Fig. 2H; Fig. S3A) and associated with decreased IL-6, CXCL-1 (KC) and macrophage inflammatory protein 1a (MIP1α) (MIP1a) production in absence of IL-1R signaling (Fig. S3B through D). Therefore, these results suggest that early IL-1R signaling is important for accumulation of ILC3s and downstream activation of myeloid cells as well as formation of protective GrALT.

To evaluate the combined importance of IL-23R and IL-1R signaling in mediating ILC3 accumulation, we utilized IL-23 neutralizing antibody either in C57BL/6 (B6) *Mtb*-infected mice or IL-23 neutralizing antibodies in *Il1r^−/−^ Mtb*-infected mice. As before (2), early IL-23 depletion in the B6 *Mtb*-infected mice resulted in increased lung *Mtb* burden as compared to isotype-treated B6 *Mtb*-infected mice at 14 dpi (Fig. 2I). However, depletion of IL-23 in *Il1r^−/−^* mice did not further increase early *Mtb* susceptibility as compared with isotype-treated *Il1r^−/−^ Mtb*-infected mice. Additionally, we did not observe any significant reduction in either ILC1 or ILC2 compartments in *Il1r^−/−^ Mtb*-infected mice that also received IL-23 neutralizing antibody as compared with the isotype-treated *Il1r^−/−^ Mtb*-infected mice (Fig. 2J and K). Indeed, we saw significant decrease in the accumulation of lung ILC3 and NKP-ILC3s in *Il1r^−/−^* mice following IL-23 neutralization at 14 dpi, suggesting that ILC3 accumulation is mediated synergistically by both IL-23/IL-1R cytokine axis (Fig. 2L and M). In addition, we observed significantly reduced AMs (Fig. 2N) as well as monocyte (Fig. 2O) accumulation following IL-23 depletion in *Il1r^−/−^ Mtb*-infected mice at 14 dpi. Therefore, our data point toward synergistic role for IL-1R/IL-23R signaling in optimal ILC3 accumulation but not ILC1 or ILC2 accumulation, and that IL-23 or IL-1R signaling alone is sufficient to mediate early *Mtb* control.

### IL1r/NFκβ signaling in lung epithelial cells regulates the accumulation of ILC3s and *Mtb* control

**Fig 3:**
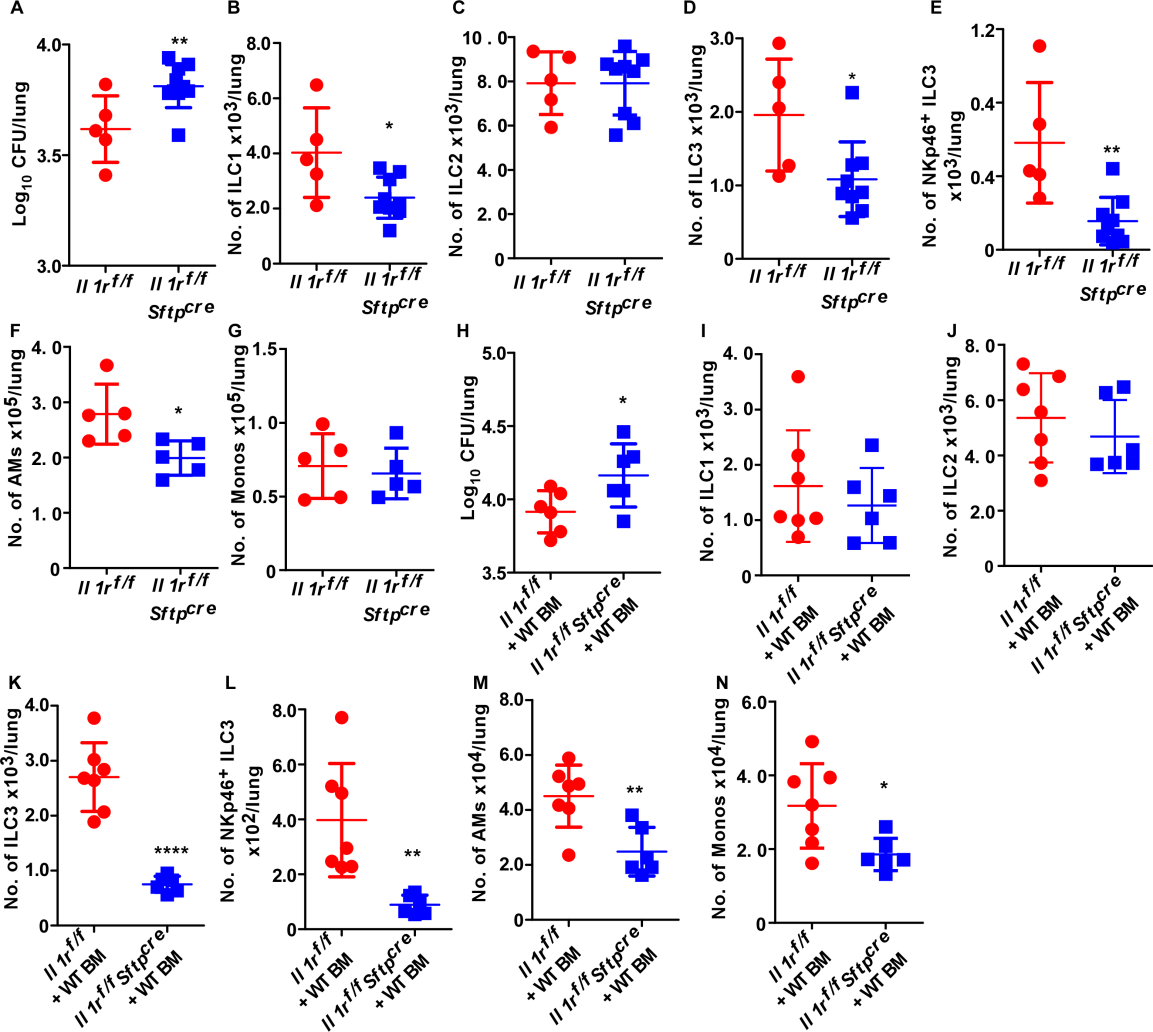
IL-1R signaling on lung epithelial cells is important for early accumulation of ILC3s following *Mtb* infection. *Il1r*^*f*/*f*^ and *Il1r^f^*^/^*^f^ Sftpc^cre^* mice were aerosol-infected with 100 CFU *Mtb* HN878. (**A**) Mice were harvested at 14 dpi, and the lung bacterial burden was determined by plating (*n* = 5–9 per group). (**B–E**) Numbers of ILC1s, ILC2s, ILC3s, and NKp46^+^ ILC3s, (**F**) AMs, and (**G**) monocytes were measured by flow cytometry on total lung single-cell suspensions at 14 dpi. In a separate experiment, *Il1r*^*f*/*f*^ and *Il1r*^*f*/*f*^*Sftpc*^cre^ mice were sublethally irradiated and reconstituted with B6 bone marrow cells. Chimeric animals were allowed to recover, then infection was done with around 100-CFU *Mtb* HN878. (**H**) Lung bacterial burden was determined by plating. (**I–N**) Numbers of ILC1s, ILC2s and ILC3s, NKp46^+^ ILC3s, AMs, and monocytes were quantified at 14 dpi using flow cytometry (*n* = 6–7 mice per group). All data are mean ± SD. *P ≤ 0.05, **P ≤ 0.01, ****P ≤ 0.0001 were determined by Student’s *t*-test (A–N).

As shown above, the IL-1/IL-23 cytokine axis regulated the accumulation of early lung ILC3s to mediate *Mtb* control. Epithelial cells are early responders to infection and induce downstream effectors such as IL-6, IL-8, TNF-α, IFN-γ, and nitric oxide (14–16). To address whether IL-1R signaling in the epithelial cells mediated downstream responses for ILC3 accumulation following *Mtb* infection, we infected *Il1r^f/f^Sftpc^cre^* (lung type 2 epithelial cells will lack IL-1R signaling) and littermate control mice with *Mtb*. Similar to *Il1r^−/−^* mice, *Il1r^f^*^/^*^f^Sftpc^cre^* mice showed early *Mtb* susceptibility with increased lung *Mtb* CFU at 14 dpi when compared to littermate control *Mtb*-infected mice (Fig. 3A). Moreover, this coincided with decreased accumulation of lung ILC1s, ILC3s, and NKP-ILC3s (Fig. 3B, D, and E), although no changes in the accumulation of lung ILC2s were noted at 14 dpi (Fig. 3C). The lung AM accumulation was significantly hampered in *Il1r^f^*^/^*^f^Sftpc^cre^* mice as compared to the *Il1r^f^*^/^*^f^ Mtb*-infected mice at 14 dpi (Fig. 3F); however, we did not observe any defects in monocyte accumulation (Fig. 3G). No differences in the baseline lung immune cell populations were observed in *Il1r^f^*^/^*^f^Sftpc^cre^* mice (Fig. S3E through M). Taken together, these data suggest that IL-1R signaling in lung type 2 epithelial cells plays an early role in accumulation of ILC3s and mediated downstream protective responses and *Mtb* control.

To further address the role played by lung epithelial cells in the early recruitment of ILC3s and *Mtb* control, we sublethally irradiated *Il1r^f^*^/^*^f^Sftpc^cre^* and littermate control mice and reconstituted both groups with B6 bone marrow (BM). After complete reconstitution (Fig. S3N), we infected the BMC (bone marrow chimeric) mice with *Mtb*. Our data show increased lung *Mtb* burden in *Il1r^f^*^/^*^f^Sftpc^cre^* recipient mice receiving wild-type BM (non-hematopoietic BMC) as compared to *Il1r^f^*^/^*^f^*recipient mice receiving B6 BM (control BMC mice) at 14 dpi (Fig. 3H). In addition, we observed reduced accumulation of lung ILC1s, ILC3s, and NKP-ILC3s (Fig. 3I, K and L), along with reduced AMs and monocytes in non-hematopoietic BMC *Mtb*-infected mice (Fig. 3M and N). As before, the accumulation of lung ILC2 was unaffected in non-hematopoietic BMC mice compared to control BMC *Mtb*-infected mice (Fig. 3J). Together, these data project an important role for IL-1R on lung epithelial cells in driving downstream accumulation of lung ILC3s and early *Mtb* control.

NF-κβ is a master regulator of lung epithelial cell-mediated immune function against pulmonary infection (17). To test if this pathway independent of IL-1R signaling is involved in accumulation of ILC3s during *Mtb* infection, we generated conditional knockout mice that lacked both IL-1R and NFκβ signaling on lung type 2 lung epithelial cells. Following *Mtb* infection, our results revealed that the *Mtb* susceptibility in the *Il1r^f^*^/^*^f^Ikk2^f^*^/^*^f^Sftpc^cre^* mice phenocopied the effect we observed in the *Il1r^f^*^/^*^f^Sftpc^cre^* mice at 14 dpi (Fig. S4A). We did not observe any further defect in ILC1 and ILC2 accumulation (Fig. S4B and C) in *Il1r^f^*^/^*^f^Ikk2^f^*^/^*^f^Sftpc^cre^* mice as compared with littermate control (*Il1r^f^*^/^*^f^Ikk2^f^*^/^*^f^) Mtb*-infected mice. However, we observed significantly reduced accumulation of ILC3 and NKP-ILC3s in *Mtb-*infected *Il1r^f^*^/^*^f^Ikk2^f^*^/^*^f^Sftpc^cre^* mice as compared with littermate control (*Il1r^f^*^/^*^f^Ikk2^f^*^/^*^f^) Mtb*-infected mice (Fig. S4D and E). We did not observe any change in AMs and monocyte population (Fig. S4F and G) at 14 dpi. Due to lack of IL-1R/NFκβ signaling, there was significantly increased lung inflammation (Fig. S4H) in *Il1r^f^*^/^*^f^Ikk2^f^*^/^*^f^Sftpc^cre^* mice as compared to *Il1r^f^*^/^*^f^Ikk2^f^*^/^*^f^* mice during *Mtb* infection. We did not detect any abnormalities in the immune cell population at baseline in both *Il1r^f^*^/^*^f^Ikk2^f^*^/^*^f^Sftpc^cre^* and littermate control mice (Fig. S4I through Q). These results support a critical role for IL-1R signaling and that the protective response driven by this pathway was NFκβ-independent.

### CXCR5 mediates ILC3 recruitment to the lung and early *Mtb* control

**Fig 4:**
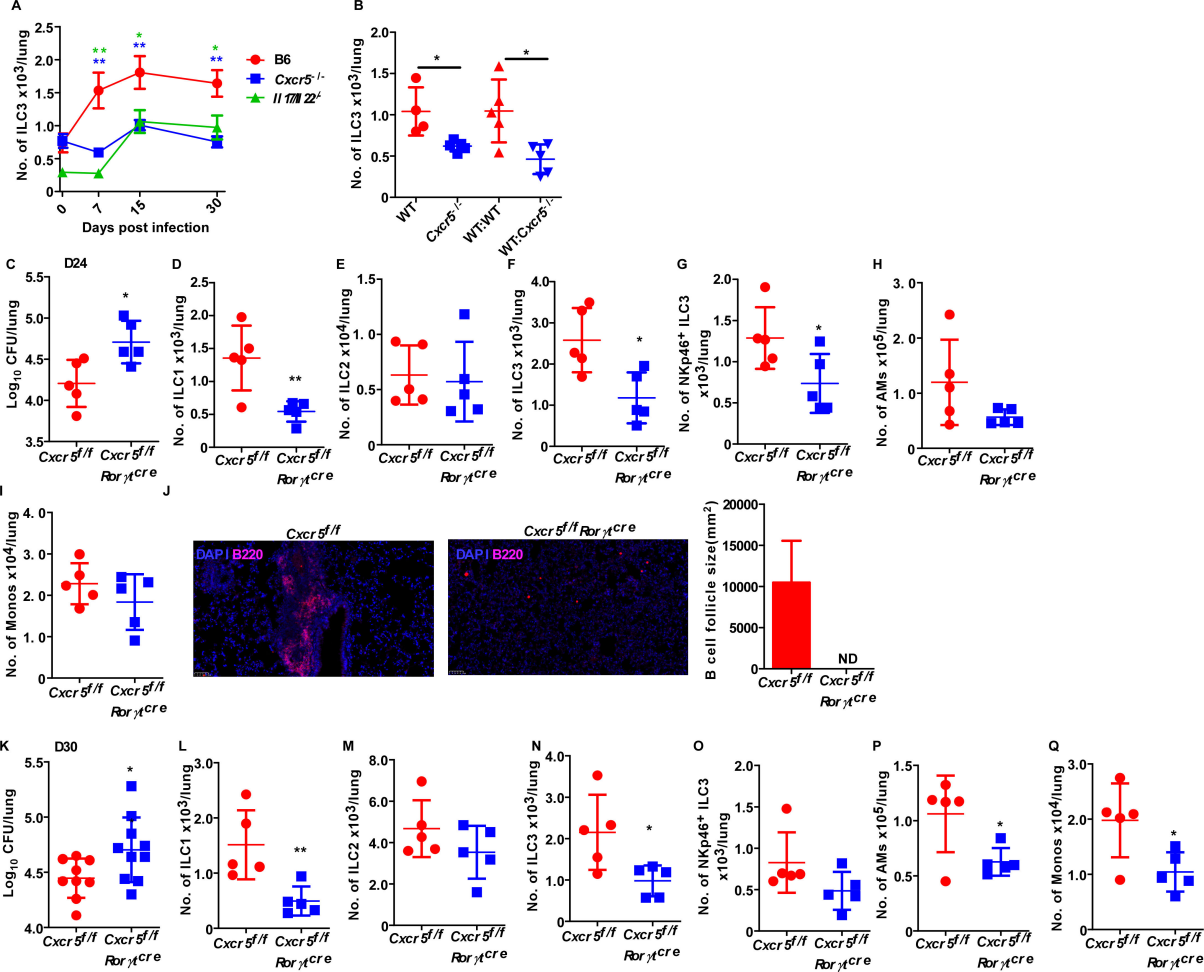
CXCR5 signaling plays critical role in ILC3 recruitment to the lung during *Mtb* infection. *Il17/22*^−/−^, *Cxcr5*^−/−^, and B6 mice were aerosol-infected with 100 CFU *Mtb* HN878. (**A**) Mice were harvested at different times post-infection, and the number of total ILC3s was measured by flow cytometry on total lung single-cell suspensions (*n* = 5–9 per group). In a separate experiment, B6 (WT) mice were sublethally irradiated and reconstituted with either B6 or *Cxcr5*^−/−^ bone marrow cells. Chimeric animals were allowed to fully reconstitute, then infection was carried out with ~100-CFU *Mtb* HN878. (**B**) Mice were harvested at 14 dpi, and the number of lung ILC3s was quantified by flow cytometry (*n* = 4–5 mice per group). *Cxcr5*^*f*/*f*^ and *Cxcr5*^*f*/*f*^*Rorγt*^cre^ mice were aerosol-infected with ~100-CFU *Mtb* HN878. (**C and K**) Lung bacterial burden at 24 and 30 dpi was determined by plating (*n* = 5–10 per group). (**D–G and L–O**) Numbers of ILC1s, ILC2s, ILC3s, and NKp46^+^ ILC3s, (**H and P**) AMs and (**I and Q**) monocytes were measured by flow cytometry on total lung single-cell suspensions at 24 and 30 dpi, respectively. (**J**) Formalin-fixed paraffin-embedded lung sections from *Mtb*-infected mice were stained with antibodies against B220, and the average sizes of B cell follicles were quantified. All data are mean ± SD. *P ≤ 0.05, **P ≤ 0.01 were determined by either two-way analysis of variance (ANOVA) (**A**), one-way ANOVA (**B**), or Student’s *t*-test (C–Q). ND, not detected.

*Cxcr5*^−/−^ mice exhibited increased lung *Mtb* CFU and decreased accumulation of ILC3s within lymphoid follicles (2). Upon kinetic analysis of the timing of lung ILC3 accumulation, deficiency of *Cxcr5* led to a delay in lung ILC3 accumulation and also resulted in fewer lung ILC3s in *Cxcr5*^−/−^ mice as compared to the control *Mtb*-infected lungs (Fig. 4A). Similar findings were observed in case of both IL-17 and IL-22 cytokines lacking (*Il17*/*Il22^−/−^*) *Mtb*-infected mice (Fig. 4A). As noted before, both *Cxcr5^−/−^* and *Il17/Il22^−/−^* mice show early *Mtb* susceptibility (2). These data therefore support a role for CXCR5 and IL-17/IL-22 in driving CXCR5 expression for early accumulation of ILC3s and *Mtb* control. To understand the role of CXCR5 in recruitment of ILC3 from circulation, we generated BMC by irradiating B6 mice and adoptively transferring BM from either B6 or *Cxcr5*^−/^*^−^* mice, allowed mice to reconstitute fully, and then *Mtb* infected the BMC mice. While the control BMC mice retained accumulation of early lung ILC3s, we observed lower accumulation of lung ILC3s in hematopoietic *Cxcr5*^−/−^ BMC mice at 14 dpi (Fig. 4B). Thus, CXCR5-mediated accumulation of ILC3s from the bone marrow is a prominent axis in accumulation of lung ILC3s to mediate *Mtb* control. To mechanistically address the role of ILC3-intrinsic CXCR5 signaling in accumulation and function of ILC3, we generated *Cxcr5^f^*^/^*^f^Rorγt^cre^* mice and infected with *Mtb*. We found that *Cxcr5^f^*^*/*^*^f^Rorγt^cre^* mice were more susceptible as compared to the littermate control mice (Fig. 4C and K) at both 24 and 30 dpi. Moreover, absence of functional CXCR5 signaling in early ILC3s resulted in decreased accumulation of ILC1, ILC3, and NKP-ILC3s (Fig. 4D, F, G, L, N, and O) in *Mtb*-infected mice lung at both time points. Similar to results in *Il1r* and IL-23 neutralized *Mtb-*infected mice, ILC2 compartment was unaltered in *Cxcr5^f^*^/^*^f^Rorγt^cre^* mice at both time points (Fig. 4E and M). Although, there was no significant defects in the AMs and monocyte accumulation at 24 dpi (Fig. 4H and I), we observed significant decrease in accumulation of AMs and monocytes (Fig. 4P and Q) in *Cxcr5^f^*^/^*^f^Rorγt^cre^* mice as compared to the littermate control mice at 30 dpi. In addition, we did not observe any GrALT formation in *Cxcr5^f^*^/^*^f^Rorγt^cre^* mice as compared with littermate control *Mtb*-infected mice at 24 dpi (Fig. 4J). No differences in the baseline lung immune cell populations were observed in *Cxcr5^f^*^*/*^*^f^Rorγt^cre^* mice (Fig. S5A through I). Thus, CXCR5 signaling in ILC3s is important for both early ILC3 accumulation, likely from the circulation and subsequent *Mtb* control.

## DISCUSSION

ILC3s are important mediators in early innate immunity against *Mtb* infection (2, 18). However, the signaling mechanisms that regulate the early recruitment and accumulation of lung ILC3s at the site of *Mtb* infection are not well defined. In the current study, we show that subsets of ILC3 and ILC3/ILC1s are increased early following *Mtb* infection, and that IL-23 and IL-1 cytokine signaling synergize to mediate early ILC3 accumulation. Furthermore, expression of CXCR5 receptor on ILC3s likely sustain lung ILC3 accumulation and mediate early *Mtb* control. Together, these findings provide detailed insights into the mechanisms that regulate ILC3 recruitment and accumulation within the lung, thereby identifying novel immune pathways that can be targeted for vaccines against TB.

Our scRNA-seq analysis revealed different clusters of ILC populations over time probably due to infection-induced changes in the microenvironment. ILC2s were the predominant lung population as reported by many other groups (11, 18, 19). Despite being the predominant ILC population, ILC2s do not play a significant role in the early protection following *Mtb* infection as IL-13 deficient mice do not show increased early susceptibility (2). The scRNA-seq analysis also identified ILC3 as well as ILC1/ILC3-like populations in the *Mtb*-infected lung. Our in depth analysis show that markers such as *Jaml*, *Gtf3a*, *Zbtb16*, *Rorc*, and *Ccr6* are upregulated in the ILC3 cluster but not in ILC3/ILC1-like cluster, while there are unique markers such as *Cxcr3* and *Lck* that are upregulated in the ILC3/ILC1-like cluster. The presence of IL-23 and IL-1R expression on ILC3s in our data set suggests that this population can directly respond to the cytokine stimuli and amplify responses following infection. However, this needs to be tested in future studies. IL23r expression within ILC3 subset has previously been reported by another group (20). Moreover, it has been shown that continuous IL-23 stimulation led to ILC3 depletion in the gastrointestinal tract (21). Therefore, the presence of this receptor could also act as a regulator of ILC3-mediated signaling during the course of infection. Interestingly, combined IL-23/TNFα stimulation led to conversion of ILC1-type population from ILC3(21). Therefore, it would be interesting to know whether lung ILC3s are also similarly responsive to cytokine stimuli and convert to another ILC population during *Mtb* infection. Corral et al. also reported the presence of ILC1-like populations which were shown to drive *Mtb*-induced type1 immune responses (18). In the gut, ILC1-like populations have been found to be derived from ILC3 populations (22). Therefore, the diversity and plasticity within the ILC populations could be attributed to the local environment and presence of microbial stimuli. ILC3 generation from ILC1-like cells likely occurs in the presence of polarizing cytokines such as IL-1β and IL-23 (23). During *Mtb* infection, we and others have reported induction of both IL-1β and IL-23 by DCs or macrophages which may lead to the conversion of ILC1s to ILC3s or, alternatively, plastic populations that express markers of both ILC1s and ILC3s. Although Corral et al. reported the presence of ILC1-like cells following *Mtb* infection (18), our results do not support a protective role for ILC1 cells in the early immunity following *Mtb* infection as *Ifnγ*^−/−^ mice did not show early innate susceptibility to *Mtb* infection (2). The discrepancy between our study, describing a protective role for ILC3s (2), and the Corral study, describing an ILC1-like cell type (18) as protective, could be due to the different infecting *Mtb* strains used. Our studies have used a clinically relevant hyper-virulent *Mtb* HN878 strain which induces more robust IL-1β responses (24, 25), as compared to lab adapted H37Rv *Mtb* strain, which was used in the Corral et al. study (18). Future work using different lineages of *Mtb* and infecting strains to study accumulation of ILC subsets in the lung following infection should shed new light on the early differentiation and protective nature of ILCs during *Mtb* infection.

One of the consistent effects of the early defect in accumulation of lung ILC3s was the decreased accumulation of AMs and monocytes in the *Mtb*-infected lung (2) [our published work (2) and this study]. The early activation of AMs has been shown to be important in their migration form the airways into the parenchyma to initiate granuloma formation (26). The localization of *Mtb-*infected AMs into the lung interstitium is also shown to be dependent on IL-1R signaling acting on lung epithelial cells (27). Additionally, in an *in vitro* co-culture system of macrophages and small airway epithelial cells, macrophages produced IL-1β in response to *Mtb* infection, which eventually acted on the epithelial cells, inducing their production of the antimicrobial peptide DEFB4/HBD2, which was effective in controlling *Mtb* replication inside the infected macrophage (14). These results are consistent with our results, suggesting that ILC3s are early innate cells that drive downstream responses to regulate AM accumulation and *Mtb* control. It is also possible that during *Mtb* infections, ILC3s can also secrete IL-1β along with other cytokines, which would then act on epithelial cells to induce downstream effector function. We also expect that *Mtb* infection-induced IL-1 will act on the ILC3s and induce ILC3 effector function or plasticity, depending on the microenvironment. Collectively, these findings point toward a novel role for ILC3s in mediating protective mechanisms by which macrophages can respond, undergo activation, and mediate early and innate *Mtb* control.

In our previous study, we demonstrated that CXCR5 signaling is important in accumulation of lung ILC3s following infection for optimal early *Mtb* control (2). Indeed, our current study confirms that CXCR5 signaling on ILC3s is important for localization of ILC3s from the periphery into the lung and supported by the increased early susceptibility seen in *Cxcr5^f^*^/^*^f^Ror^cre^ Mtb*-infected mice. Lung ILC3s are likely the first responder cells following *Mtb* infection and CXCR5^+^ ILC3s likely accumulate in the lung from the periphery in response to *Mtb* infection. We have not observed any defects in terms of *Mtb* clearance in *Cxcr5^f^*^/^*^f^Ror^cre^* mice at 15 dpi. However, we did find lower accumulation of ILC3s at 15 dpi in *Cxcr5^f^*^/^*^f^Ror^cre^* mice, with increased susceptibility at 24 dpi. Therefore, we speculate that the resident ILC3s and local proliferation of these resident cells are enough to provide early immunity following *Mtb* infection, while CXCR5-expressing ILC3s may be recruited from the periphery and contribute to early but sequential protection at 24 dpi. The ligand for CXCR5, namely, CXCL13, is inducible in the murine lung following infection with *Mtb* (28–30). In our previous study, we demonstrated that mouse and human ILCs migrate in response to CXCL13 in a CXCR5-dependent manner (2). Epithelial cells are a potential source of CXCL13 as reported by us and many other groups (31, 32). Therefore, it is possible that the early induction of CXCL13 within lungs of *Mtb*-infected mice is responsible for the recruitment of CXCR5-expressing ILC3s from circulation. Furthermore, in our previous study, we demonstrated that *Il17/Il22*^−/−^ double-knockout mice displayed a significant early increase in lung *Mtb* burden, decreased number of lung ILC3s as well as CXCR5-expressing ILC3s, and decreased expression of *Cxcl13* mRNA within GrALT (2). Therefore, further detailed mechanistic analysis of the distinct ILC3 subsets in the future will provide additional insights into understanding the role of lung versus recruited populations of ILC3s in *Mtb* infection, and other pulmonary infections.

Additionally, ILC3s can present antigen to T cells, thereby helping in further T-cell activation (33), and ILC3s are known to interact with B cells (34). ILC3s can control B-cell function and homeostasis in a T cell-independent manner. Human, (RORγt)+ ILCs (mostly ILC3s) activate naïve, marginal zone, and plasma B cells by expressing cell surface molecules such as CD40L, and Delta-like 2 (a notch ligand)(35), thereby further inducing IgA-class switching in lymphoid structures (36). Our scRNA-seq showed the expression of *Cd40lg* in ILC3 clusters, likely induced due to ongoing infection. Therefore, it is also possible that the ILC3s play an early role in activation and accumulation of T and B cells and formation of protective GrALT. Recently, another report described intestinal “trained” ILC3s (Tr-ILC3) that emerged and persisted after initial encounter with *Citrobacter rodentium (37*). Further infection led to Tr-ILC3s proliferation and robust production of IL-22, thus promoting mucosal defense. Therefore, it is exciting to consider that ILC3s can be targeted for activation by adjuvants for vaccine design against TB.

In summary, our data revealed *Mtb* infection-induced ILC populations, especially ILC3 subsets, which are potentially being regulated by the lung epithelial signaling. Moreover, we demonstrated novel role played by IL-1, IL-23, and CXCR5 signaling in ILC3 accumulation within *Mtb-*infected mice lung. Therefore, understanding the early signaling events responsible for ILC3-mediated immune response will help in the development of targeted therapeutic strategies aimed at enhancing ILC3-mediated protection against *Mtb* infection.

## MATERIALS AND METHODS

### Mice

C57BL/6 (B6), B6.129S7-Il1r1^tm1Imx^/J (*Il1r*^−/−^), and B6.129(Cg)-Il1r1^tm1.1Rbl^/J (Il1r^f*/*f^) mice were obtained from Jackson Laboratory (Bar Harbor, ME) and bred at Washington University in St. Louis. *Il17^−/−^* ^(^38^)^ and *Il22^−/−^* ^(^39^)^ mice were crossed at Washington University in St Louis to obtain double knockout mice. *Cxcr5^f^*^/^*^f^* mice were generously donated by Dr. Nell Mabbott and Dr. Barry Bradford from University of Edinburgh. *Ikk2^f^*^/^*^f^ Sftpc^cre^* mice were a kind gift from Dr. Pasparakis (University of Cologne). Mice were age- and sex-matched and used between 6 and 8 weeks of age. All mice were used and housed in accordance with the National Institutes of Health guidelines for housing and care of laboratory animals. *Il1r^f^*^/^*^f^ Sftpc^cre^* mice were obtained by crossing the *Il1r^f^*^/^*^f^* strain with *Sftpc^cre^* mice. Similarly, *Il1r^f^*^/^*^f^ Ikk2^f^*^/^*^f^ Sftpc^cre^* and *Cxcr5^f^*^/^*^f^Rorγt^cre^* mice were generated.

### IL-23 *in vivo* neutralization

Anti-IL-23 (Amgen, 16E5, 500 µg per mouse) and mouse IgG1 isotype (500 µg per mouse) were provided by Amgen and intraperitoneally injected into C57BL/6 mice 1 day before infection.

### Bacterial infection

*Mtb* W. Beijing strain, HN878 (BEI Resources), were grown to mid-log phase in Proskauer Beck medium containing 0.05% Tween 80 and frozen in at −80°C. Mice were infected using a Glas-Col airborne infection system, with approximately 100 CFU of bacteria as described previously (40). Lungs were collected and homogenized; serial dilutions of tissue homogenates were plated on 7H11 plates; and the CFU was counted. At given time points following infection, lungs were collected and homogenized, and the tissue homogenates were plated following serial dilutions on 7H11 agar (BD Bioscience) to assess bacterial burden.

### Generation of single-cell suspensions from tissues

Lung single-cell suspensions from *Mtb*-infected mice were isolated as previously described (41). Briefly, mice were euthanized with CO_2_, and lungs were perfused with heparin in saline. The lungs were minced and incubated in Collagenase/DNAse (Sigma-Aldrich) for 30 minutes at 37°C. Lung tissue was pushed through a 70-µm nylon screen to obtain a single-cell suspension. Red blood cells were lysed, and the cells were re-suspended in suitable media or buffer for further use.

### Generation of bone marrow chimera

Recipient *Il1r^f^*^/^*^f^*, *Il1r^f^*^/^*^f^ Sftpc^cre^*, or B6 mice were exposed to two doses of 450 cGy of X-rays in 48 hours. Following irradiation, mice were rested for 6–8 hours before donor’s bone marrow cell transplant. Naïve B6 or *Cxcr5*^−/−^ mice euthanized, and cells were isolated from the femur and tibia. Bone marrow cells (1 × 10^7^) were transplanted into the recipient mice by intravenous route, and the mice were put on antibiotic-water (sulfamethoxazole and trimethoprim oral suspension, NDC 62559–550-16). After 9 weeks, the reconstitution efficacy was checked via flow cytometry, and the chimeric mice were infected with *Mtb* as described before.

### ILC cell sort

ILC populations were sorted from mice infected with HN878 *Mtb* at 5 and 14 dpi. At the indicated time post-infection, mice were sacrificed, and lung single-cell suspensions were made as previously described (2). CD45^+^ immune cells populations were first enriched from mice using CD45^+^ microbeads according to the manufacturer’s instruction (Miltenyi Biotec). These enriched populations were then stained with the following antibody cocktail: CD45, CD127, NK1.1, and lineage markers (CD3, CD5, CD19, TER119, CD11b, and CD11c). To isolate out the ILC population, the Lin^−^NK1.1^−^CD45^+^CD127^+^ cells were sorted using the BD FACS Jazz flow cytometer with FACS Sortware software (BD). The purity of the sorted population was analyzed and reported to be >98% (Fig. S1A). Cells were collected into phosphate-buffered saline containing 0.04% non-acetylated bovine serum albumin and were subjected to scRNA seq.

### scRNA-seq library generation and sequencing

Highly pure ILC populations were sorted as mentioned before and subjected to droplet-based, massively parallel single-cell RNA sequencing using a Chromium Single Cell 3′ (v.3) reagent kit according to the manufacturer’s instructions (10× Genomics) as described before (41). Briefly, cell suspensions were loaded at 1,000 cells/µL to capture 10,000 cells/lane. The 10× Chromium Controller generated gel bead-in-emuslion (GEM) droplets, where each cell was labeled with a specific barcode, and each transcript was labeled with a unique molecular identifier (UMI) during reverse transcription. The barcoded cDNA was isolated and removed from the BSL-3 space for library generation. The cDNA underwent 11 cycles of amplification, followed by fragmentation, end repair, A-tailing, adapter ligation, and sample index PCR according to the manufacturer’s instructions. Libraries were sequenced on a NovaSeq S4 (200 cycle) flow cell, targeting 50,000 read pairs/cell.

### scRNA-seq data processing

The raw gene expression matrices were generated by the Cell Ranger software (10× Genomics, v.3) available on the 10× website. After demultiplexing, the resulting fastq files were aligned against the mouse reference genome mm10 with cellranger count. For each sample, the recovered-cell parameter was set to 10,000 cells that we expect to recover for each library. The output filtered gene count matrices were analyzed by R software (v.4.0.4) with the Seurat (42) package (v.4.1.0). Cells that had less than 600 and more than 2,200 detected genes were filtered out. Different thresholds were chosen for mitochondrial genes because of their distinct distribution in each sample. Criteria for filtered cells were as follows: (i) had more than 6% (D5) and (ii) 5% (D14) of mitochondrial genes. Samples were merged and normalized with default parameters, and most variable genes were detected by the FindVariableFeatures function. ScaleData was used to regress out the number of UMIs and mitochondrial content, and principal component analysis (PCA) was performed with RunPCA. The *t*-distributed stochastic neighbor embedding (tSNE) dimensionality reduction was performed on the scaled matrix using the first 18 PCA. For clustering, the FindNeighbors (18 PCA) and FindClusters (resolution 0.5) functions were used. FindAllMarkers was used to compare a cluster against all other clusters to identify the marker genes. For each cluster, the minimum required average log fold change in gene expression was set to 0.25, and the minimum percentage of cells that must express gene in either cluster was set to 25%. To re-analyze ILC populations, we pooled the clusters that we identified as ILC origin and re-run PCA, tSNE, and clustering (resolution 1) to get a better resolution for further analysis.

### Flow cytometry staining

The following antibodies were from TonBo Biosciences: CD127 (clone A7R34), CD3 (clone 145–2C11), and CD19 (clone 1D3). Antibodies purchased from eBioscience were RORc(γt) (clone AFKJS-9) and Sca-I (clone D7). CD45 (clone 30-F11) and streptavidin were purchased from Becton Dickinson. The following antibodies were from BioLegend: biotinylated NKp46, TER-119 (clone TER-119), CD11c (clone N418), CD5 (clone 53–7.3) and CD11b (clone M1/70). Gr1 (clone RB6-8C5) and NK1.1 (clone PK136) were purchased from BD Bioscience. Live/dead AQUA was purchased from ThermoFisher Scientific. For intracellular staining, fixation/permeabilization concentrate and diluent (eBioscience) were used to fix and permeabilize lung cells for 20 min. The cells were incubated overnight with intracellular staining antibodies. Samples were run on a four-laser BD Fortessa flow cytometer. All flow cytometry data were analyzed using FlowJo (v.9.7.6, TreeStar).

### Histological analysis

The right upper lobe was collected at the time of harvest for histological analysis of inflammation as previously described. Briefly, the lobes were perfused with 10% neutral buffered formalin and embedded in paraffin. Sections (5  µm) of formalin-fixed and paraffin-embedded (FFPE) lung were cut with a microtome, stained with hematoxylin and eosin, and processed for light microscopy. Images were captured using the automated NanoZoomer digital whole-slide imaging system (Hamamatsu Photonics). Regions of inflammatory cell infiltration were delineated utilizing the NDP view2 software (Hamamatsu Photonics), and the total area of inflammation was calculated in individual lung lobes by adding all the inflammatory regions of individual lung lobes. All scoring was conducted in a blinded manner utilizing *n* = 5–10 mice per group.

### Immunofluorescence staining

Immunofluorescence staining was done as described before (2). FFPE lung sections were cut, immersed in xylene, and then hydrated in alcohol and PBS. Antigens were unmasked using DakoCytomation target retrieval solution (Dako), and non-specific binding was blocked by adding 5% (vol/vol) normal donkey serum and Fc block (BD). Avidin was used to neutralize endogenous biotin, followed by incubation with biotin (Sigma-Aldrich). Sections were then probed with anti-B220 (clone RA3-6B2, BD) to detect B cells. For analysis of B-cell follicles, follicles were outlined with an automated tool of the Zeiss Axioplan 2 microscope (Zeiss), and total area and average size were calculated in squared microns.

### Cytokine and chemokine quantification using Luminex or ELISA

Cytokine and chemokine protein contents in lung homogenates were quantified using Luminex multianalyte technology (Millipore) according to the manufacturer’s protocols.

### Statistical analysis

The differences between the two groups were analyzed using two-tailed Student’s *t*-test in Prism (v.9, GraphPad). Differences between the means of three or more groups were analyzed using one-way analysis of variance (ANOVA) with Tukey’s post-test. For comparisons between two or more groups with two independent variables, two-way ANOVA with Sidak’s or Tukey’s post-test was used. A *P* value of <0.05 was considered significant.

## Data Availability

All relevant data are available from the authors. The GEO accession number for the raw and processed data for sc-RNA seq generated during this study is GSE249667.

## SUPPLEMENTAL MATERIAL

Figure S1 mbio.03299-23-s0001.pdf Hierarchical gating strategy used to identify immune cell populations in mouse lungs.

Figure S2 mbio.03299-23-s0002.tif scRNA-seq transcriptional profiling of lung ILCs isolated following from *Mtb* infection.

Figure S3 mbio.03299-23-s0003.tif Absence of IL-1R signaling in lung epithelial cells does not impact baseline immune cells in naïve mice.

Figure S4 mbio.03299-23-s0004.tif Absence of IL-1R signaling and NFKB in lung epithelial cells does not impact baseline immune cells in naïve mice.

Figure S5 mbio.03299-23-s0005.tif Absence of CXCR5 signaling in ILC3s does not impact baseline immune cell numbers in the lung.

Legends mbio.03299-23-s0006.docx Supplemental figure legends.

ASM does not own the copyrights to Supplemental Material that may be linked to, or accessed through, an article. The authors have granted ASM a non-exclusive, world-wide license to publish the Supplemental Material files. Please contact the corresponding author directly for reuse.
