# Supplementary figures and images for: Lung type 3 innate lymphoid cells respond early following Mycobacterium tuberculosis infection
Source: mBio. 2024 Feb 26;15(4):e03299-23. doi: 10.1128/mbio.03299-23 (PMC11005430; doi:10.1128/mbio.03299-23)

A

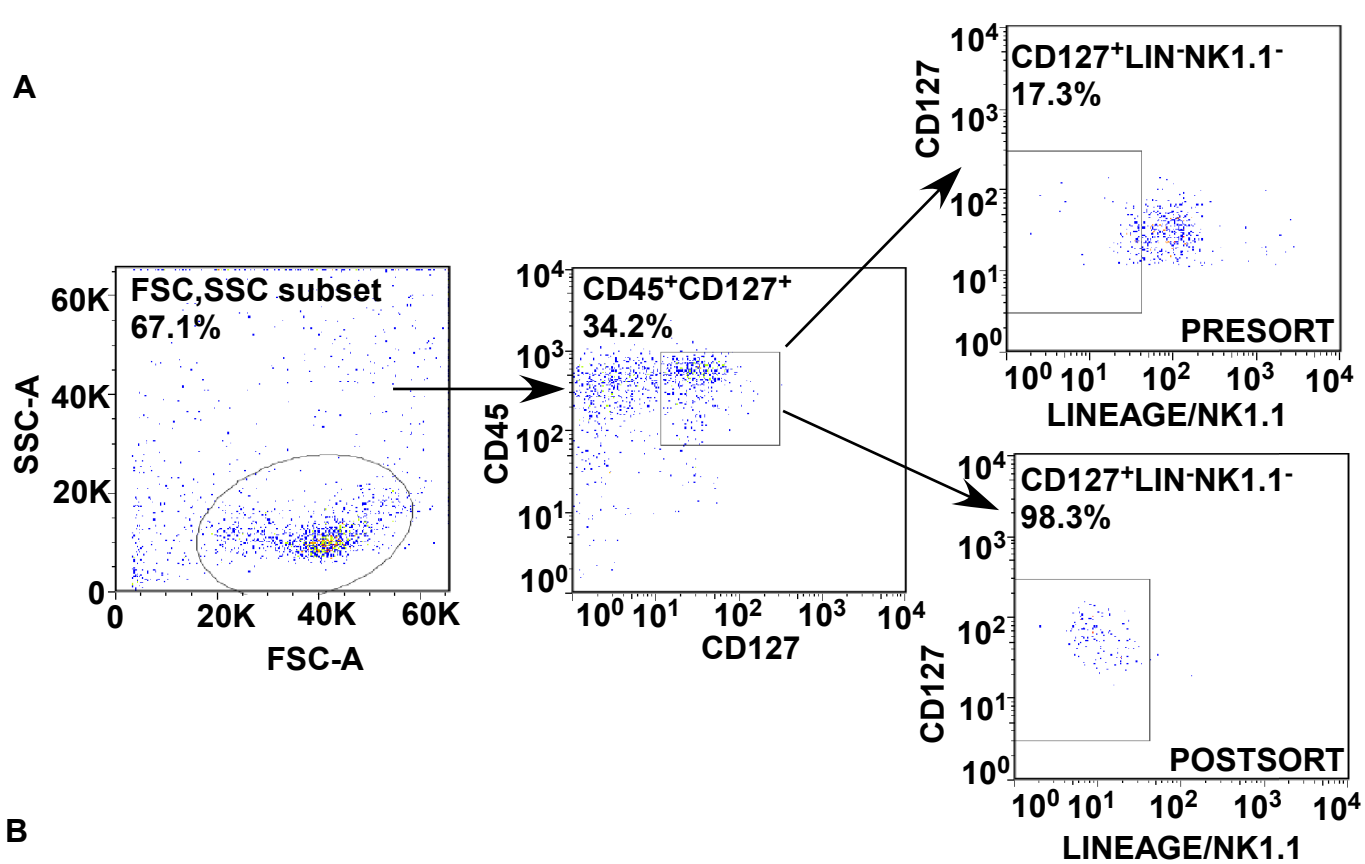

B

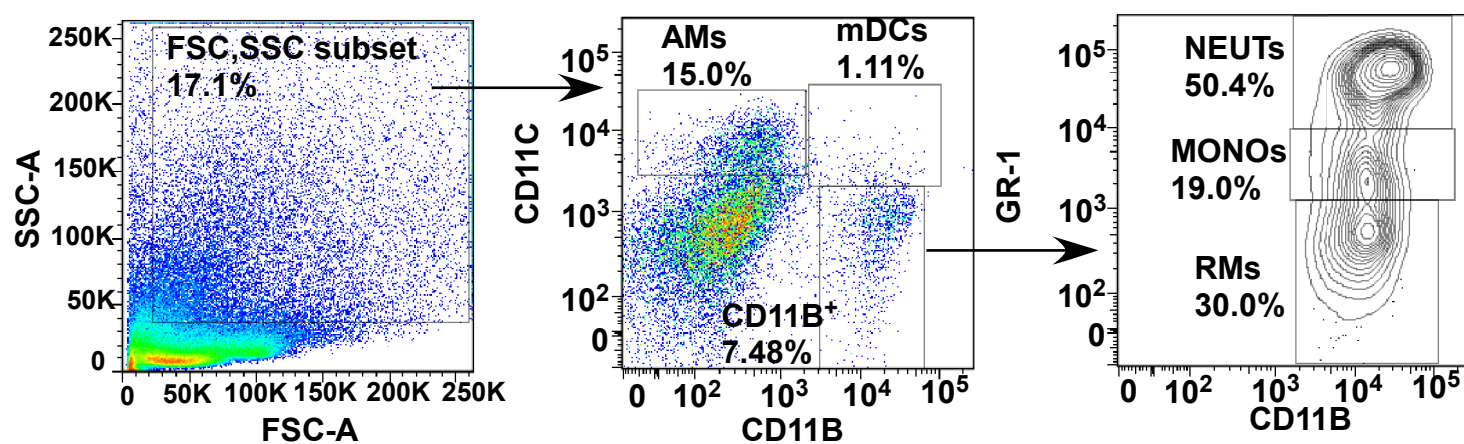

C

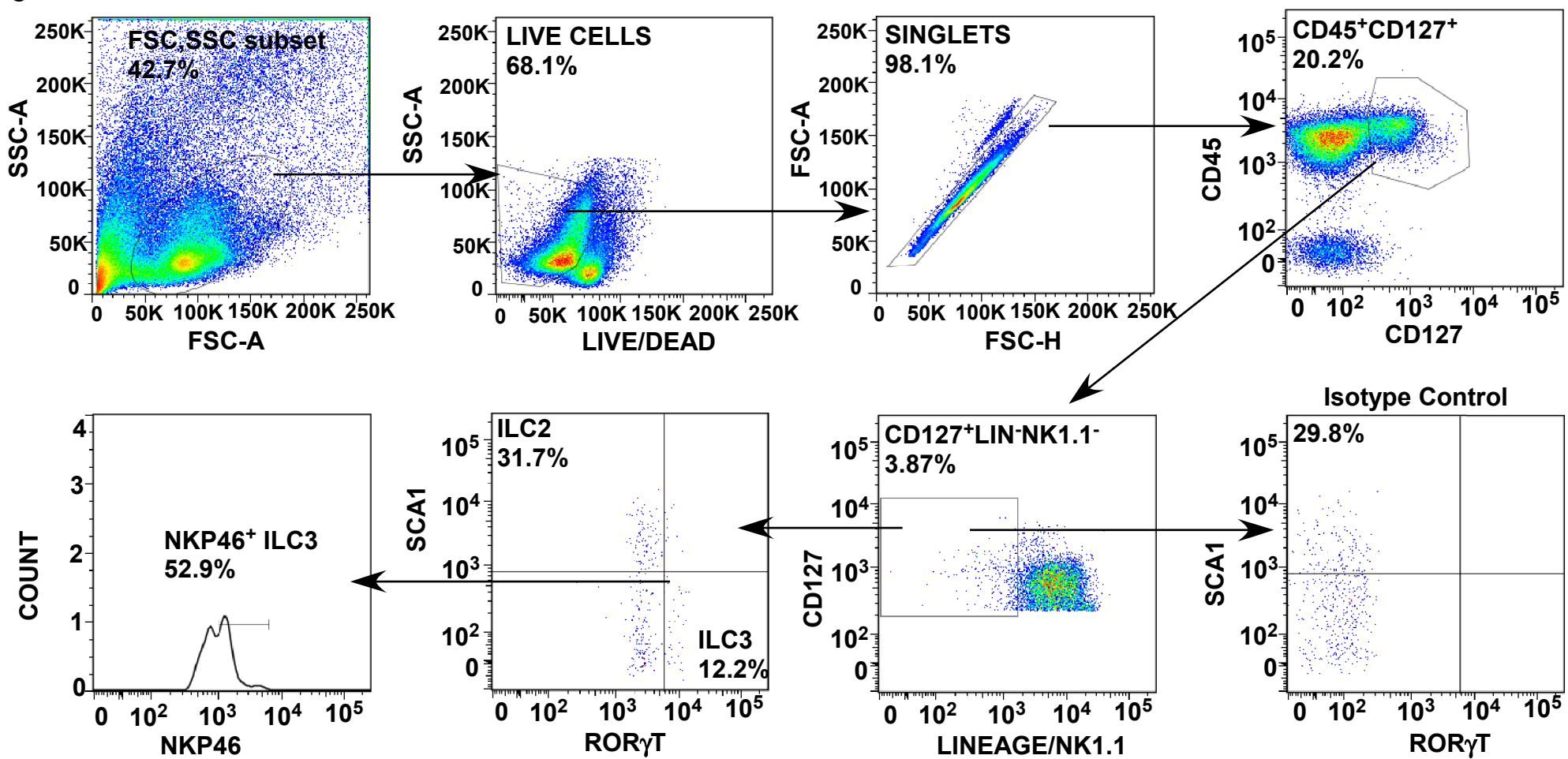

D

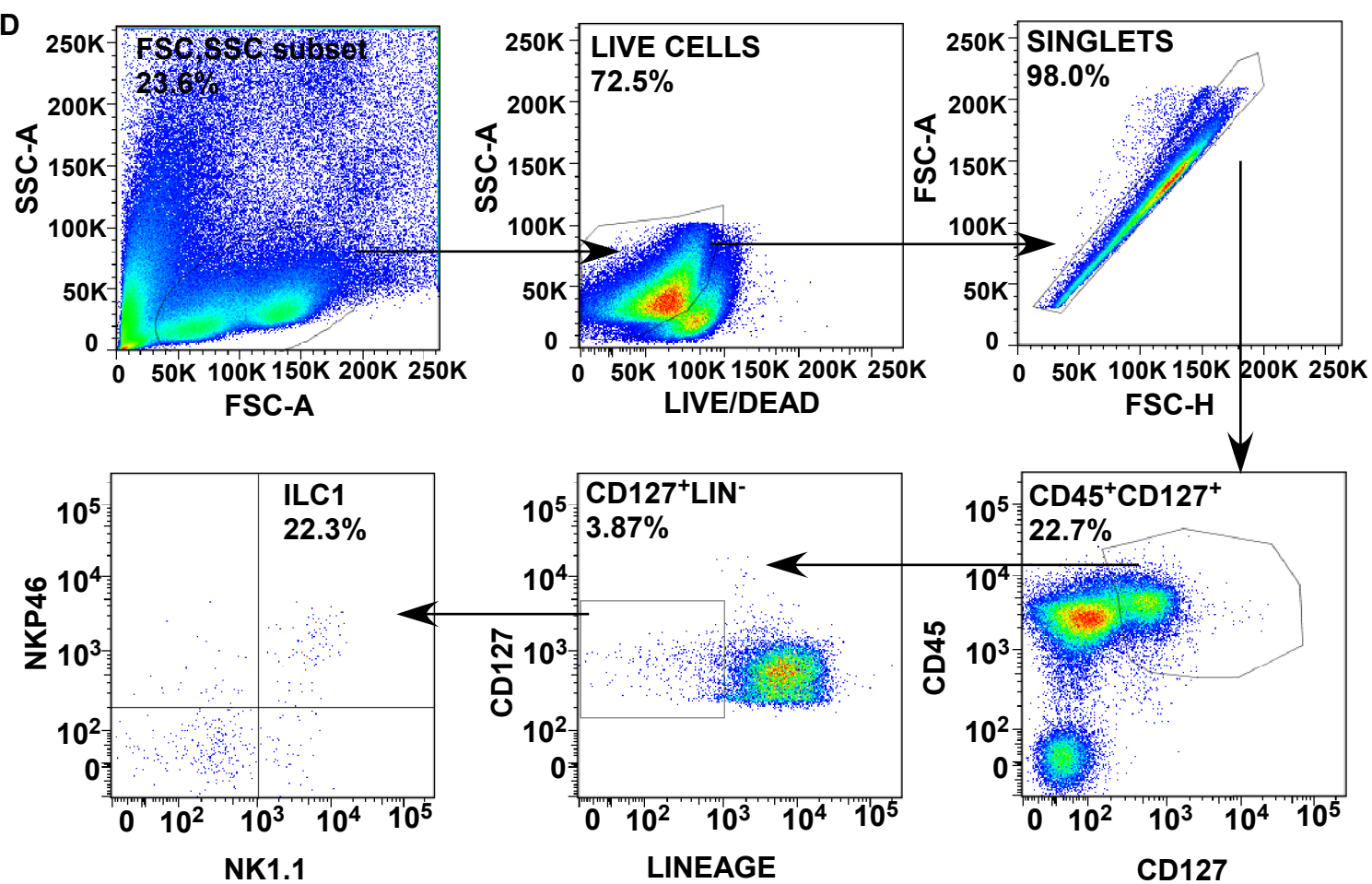

Supplement: Figure S1 — Hierarchical gating strategy used to identify immune cell populations in mouse lungs. [file mbio.03299-23-s0001.pdf]

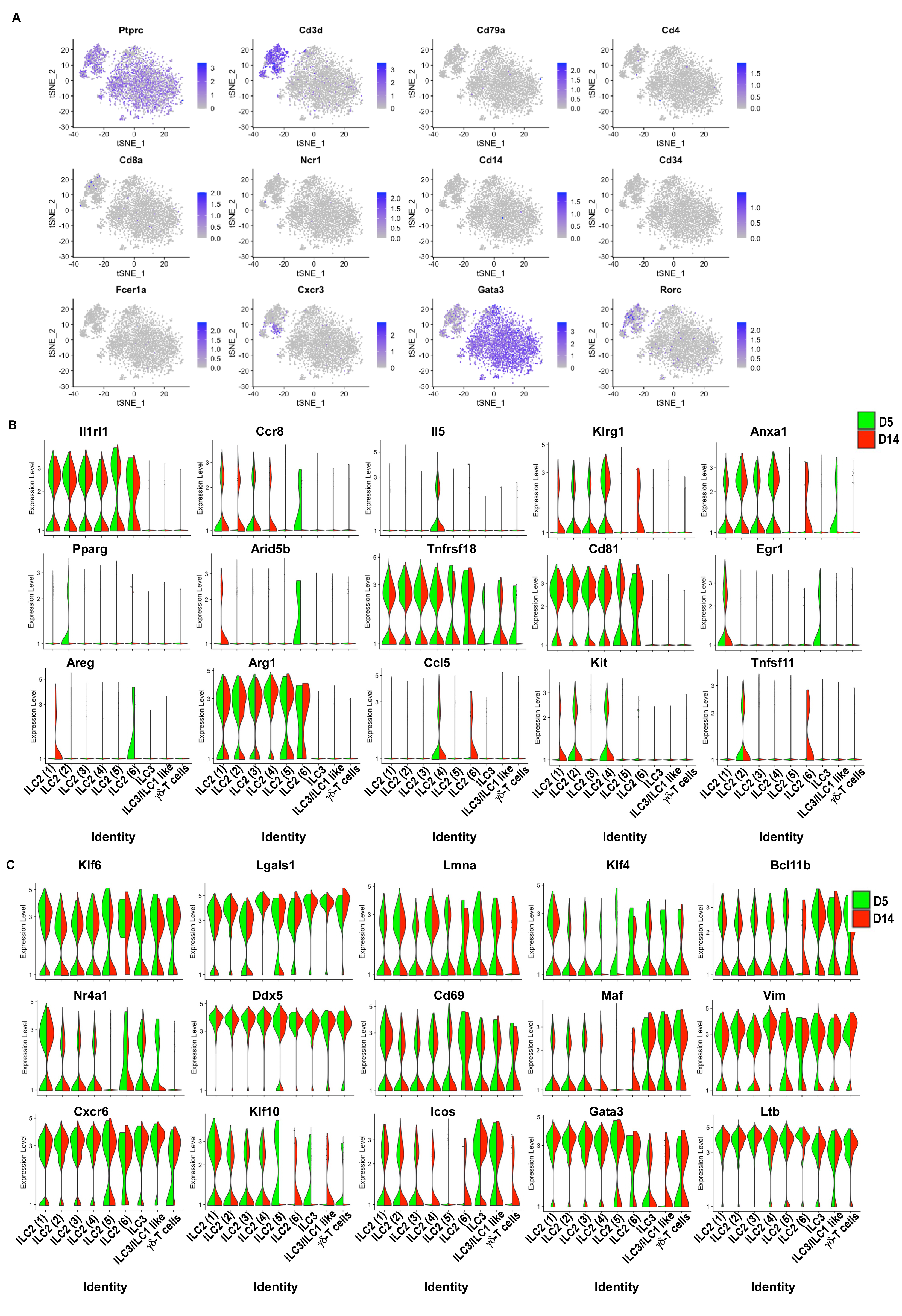

Supplement: Figure S2 — scRNA-seq transcriptional profiling of lung ILCs isolated following from Mtb infection. [file mbio.03299-23-s0002.tif]

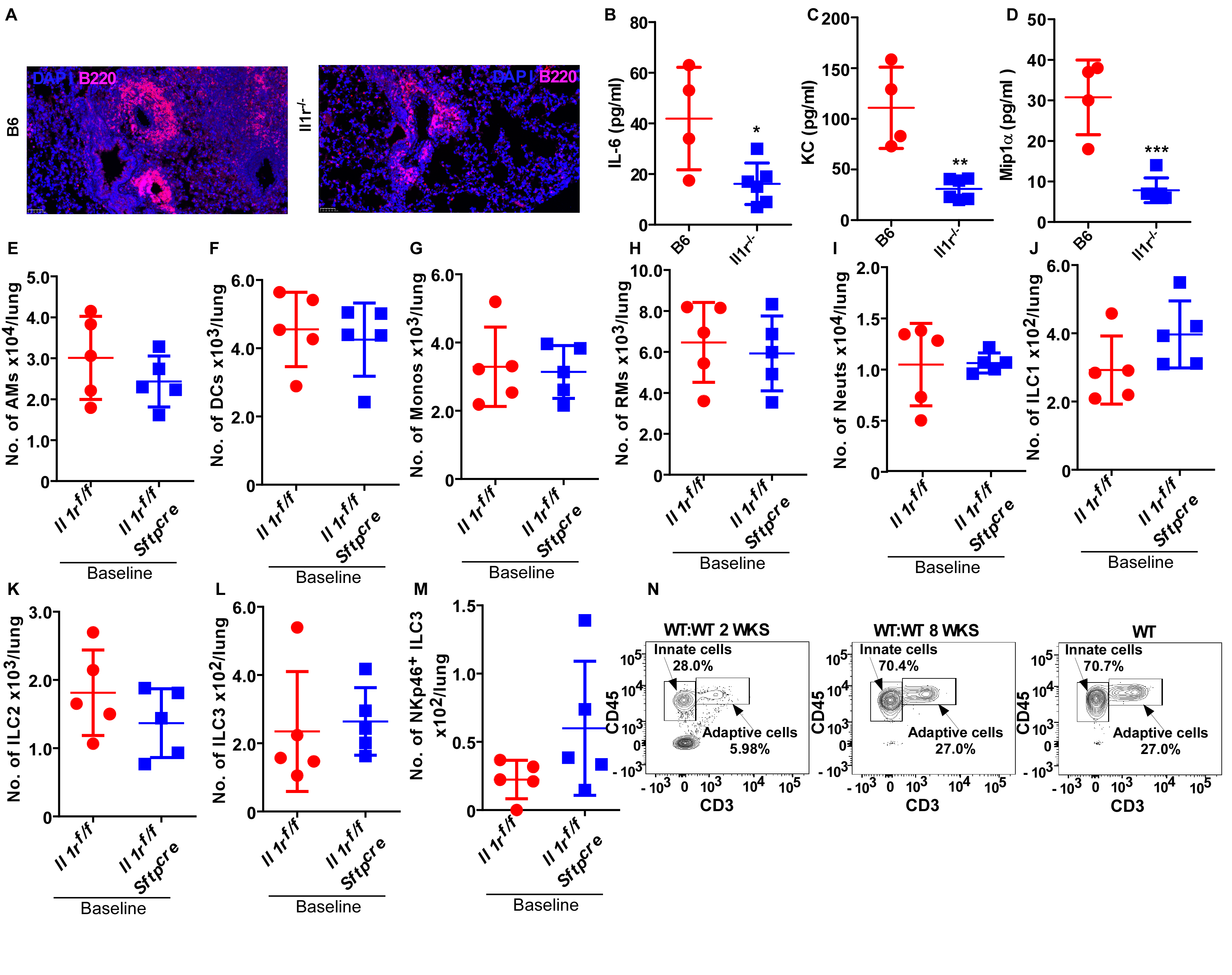

Supplement: Figure S3 — Absence of IL-1R signaling in lung epithelial cells does not impact baseline immune cells in naïve mice. [file mbio.03299-23-s0003.tif]

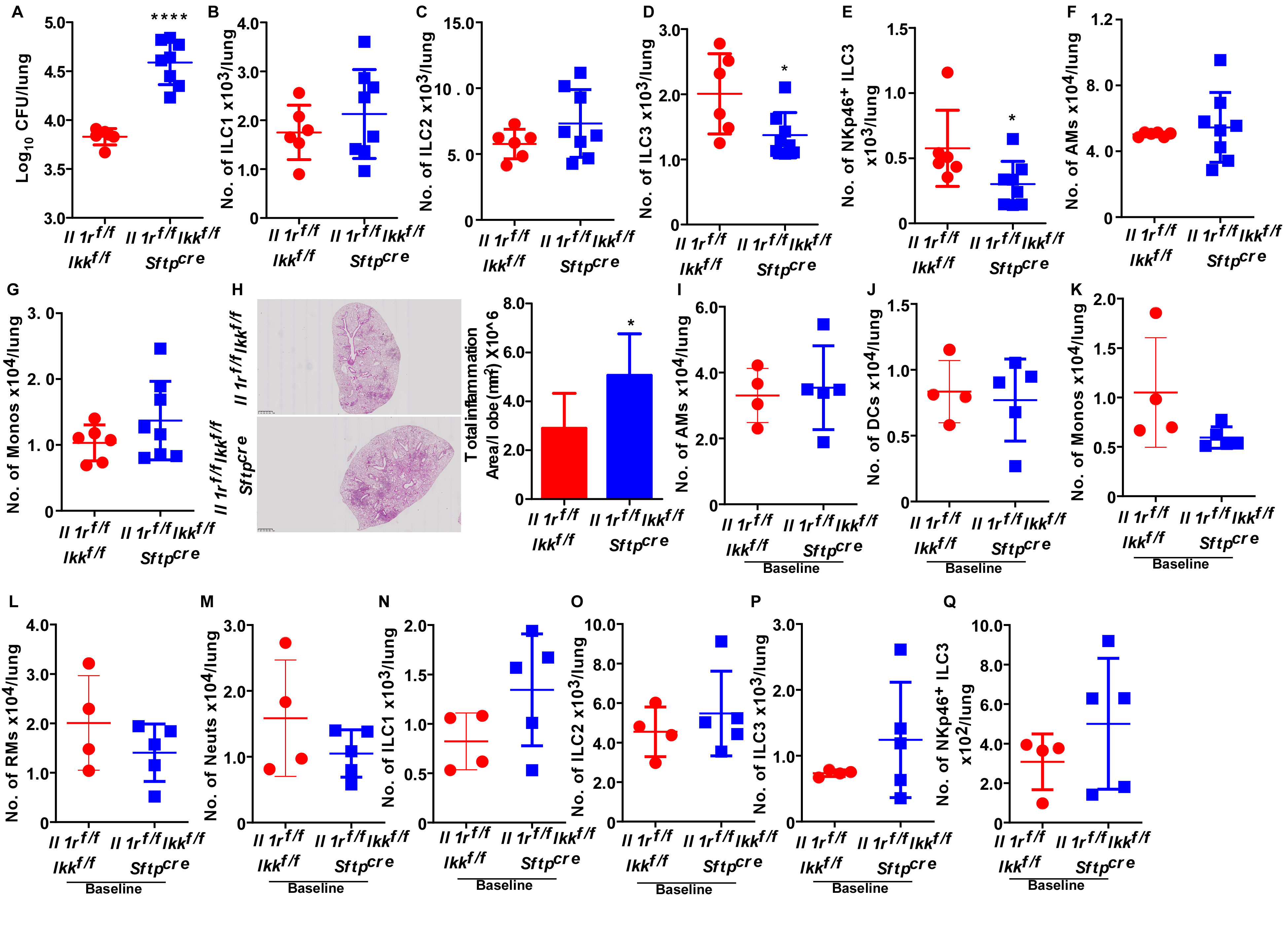

Supplement: Figure S4 — Absence of IL-1R signaling and NFKB in lung epithelial cells does not impact baseline immune cells in naïve mice. [file mbio.03299-23-s0004.tif]

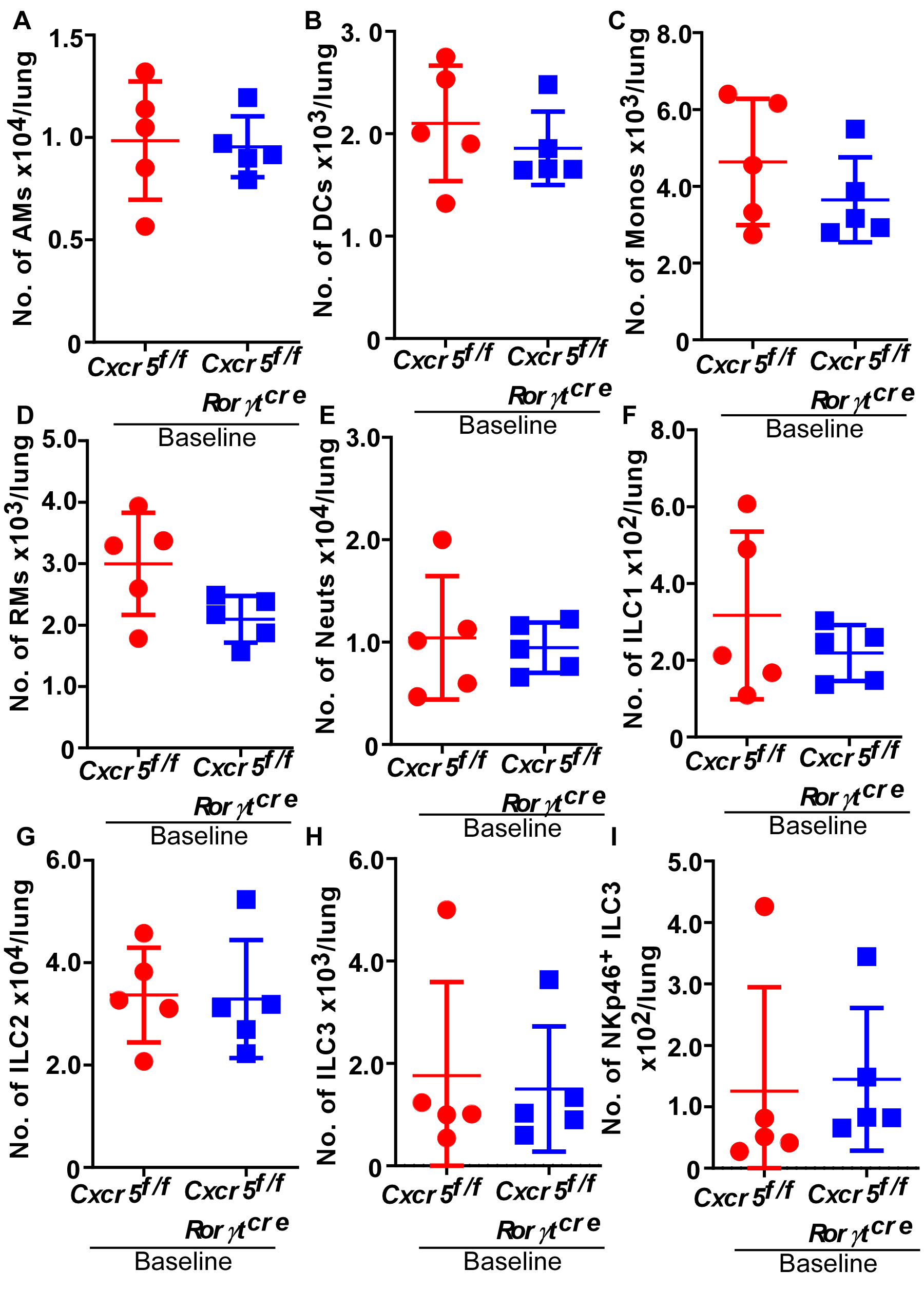

Supplement: Figure S5 — Absence of CXCR5 signaling in ILC3s does not impact baseline immune cell numbers in the lung. [file mbio.03299-23-s0005.tif]
