# Supplementary material for: Lung type 3 innate lymphoid cells respond early following Mycobacterium tuberculosis infection
Source: mBio. 2024 Feb 26;15(4):e03299-23. doi: 10.1128/mbio.03299-23 (PMC11005430; doi:10.1128/mbio.03299-23)
Supplement: Legends — Supplemental figure legends. [file mbio.03299-23-s0006.docx]

***Supplementary Figure 1. Hierarchical gating strategy used to identify immune cell populations in mouse lungs. B***6 mice were aerosol infected with ~100 CFU *Mtb* and lungs were collected at different dpi and flow cytometry analysis was carried out on single-cell suspensions to identify different immune cell populations. (**A**) ILCs were sorted from *Mtb* infected lung and reflowed back into FACSJazz to confirm purity. Purity of ILCs was confirmed to be greater than 98%. (**B**) Flow gating strategies for lung myeloid cell population from uninfected or *Mtb*-infected mice: AMs (CD11c^+^CD11b^−^), mDCs (CD11b^+^CD11c^+^), Neuts (CD11b^+^CD11c^-^Gr1^hi^), monocytes (CD11b^+^CD11c^-^Gr1^lo^) and RMs (CD11b^+^CD11b^-^Gr1^-^) are shown. (**C**) Flow gating strategies for ILC2s (CD45^+^CD127^+^Lin^−^NK1.1^−^Sca1^+^) and ILC3 (CD45^+^CD127^+^Lin^−^NK1.1^−^Rorγt^+^) and NKp46-expressing (CD45^+^CD127^+^Lin^−^NK1.1^−^Rorγt^+^NKp46^+^) ILC3s are shown.  (**D**) Flow gating strategies for ILC1s (CD45^+^CD127^+^Lin^−^NKp46^+^NK1.1^+^) are shown.

***Supplementary Figure 2*.** **scRNA-seq transcriptional profiling of lung ILCs isolated following from *Mtb* infection.** B6 mice were aerosol infected with *Mtb* HN878 and lungs were harvested at 5 and 14 dpi. (**A**) tSNE plot with the expression of known cell markers. The expression of marker genes was used to characterize distinct clusters according to cell identity. (**B,C**) Violin plot of genes in ILC subsets by cluster per days (infected at 5 dpi, pooled from 10 mice; infected at 14 dpi, pooled from 10 mice).

***Supplementary Figure 3: Absence of IL-1R signaling in lung epithelial cells does not impact baseline immune cells in naïve mice.*** *Il1r*^−/−^ and B6 mice were aerosol-infected with ~100 CFU *Mtb* HN878. (**A**) Formalin-fixed paraffin-embedded lung sections from *Mtb*-infected mice were stained with antibodies against B220, and representative image is shown. (**B-D**) Mice were harvested at 14 dpi and the levels of IL-6, KC and Mip-1α in lung homogenates were quantified by luminex multiplex assay (*n* = 4-6 per group). Naïve *Il1r*^f/f^ and *Il1r*^f/f^ *Sftpc*^cre^ mice were sacrificed and total lung single cell suspension were used to analyze immune cells at baseline. (**E**-**M)** Number of AMs, Dendritic cells (DCs), monocytes, recruited macrophages (RMs), neutrophils, ILC1s, ILC2s, total ILC3s and NKp46^+^ ILC3s were measured by flow cytometry on total lung single cell suspensions  (*n* = 5 mice per group). (**N**) B6 mice were sub lethally irradiated and reconstituted with B6 bone marrow cells. Chimeric animals were allowed to recover and the peripheral blood was subjected to flow cytometry to check reconstitution efficiency in recipient mice. All data are mean ± s.d. *, P ≤ 0.05; **, P ≤ 0.01; ***, P ≤ 0.001 were determined by Student’s *t-*test.

***Supplementary Figure 4. Absence of IL-1R signaling and NFKB in lung epithelial cells does not impact baseline immune cells in naïve mice.*** *Il1r*^f/f^*Ikk2*^f/f^ and *Il1r*^f/f^*Ikk2*^f/f^ *Sftpc^cre^* mice were aerosol-infected with ~100 CFU *Mtb* HN878. (**A**) Mice were harvested at 14 dpi, and the lung bacterial burden was determined by plating (*n* = 6-8 per group). (**B**-**E)** Number of ILC1s, ILC2s, ILC3s and NKp46^+^ ILC3s; (**F**) AMs and (**G**) monocytes were measured by flow cytometry on total lung single cell suspensions. (**H**) Lung covered by inflammation on Hematoxylin & Eosin–stained formalin-fixed paraffin-embedded (FFPE) sections was quantified using the morphometric tool of the Nanozoomer microscope. In a separate experiment, naïve *Il1r^f/f^Ikk2^f/f^* and *Il1r*^f/f^*Ikk2*^f/f^*Sftpc^cre^* mice were harvested and total lung single cell suspension were made to analyze baseline immune cell numbers. (**I**-**Q)** Number of AMs, DCs, monocytes, RMs, neutrophils, ILC1s, ILC2s, total ILC3s and NKp46^+^ ILC3s were measured by flow cytometry on total lung single cell suspensions.  (*n* = 4-5 mice per group).  All data are mean ± s.d. *, P ≤ 0.05; ****, P ≤ 0.0001 were determined by Student’s *t-*test.

***Supplementary Figure 5. Absence of CXCR5 signaling in ILC3s does not impact baseline immune cell numbers in the lung.*** Naïve *Cxcr5^f/f^* and *Cxcr5^f/f^* *Rorγt*^cre^ mice were harvested and total lung single cell suspension were made to analyze immune cell infiltration. (**A**-**I)** Number of AMs, DCs, monocytes, RMs, neutrophils, ILC1s, ILC2s, ILC3s and NKp46^+^ ILC3s were measured by flow cytometry on total lung single cell suspensions.  (*n* = 5 mice per group).  All data are mean ± s.d. Significance were determined by Student’s *t-*test.
